# How to measure diffusion coefficients in biofilms: A critical analysis

**DOI:** 10.1002/bit.27650

**Published:** 2020-12-25

**Authors:** Lenno van den Berg, Mark C. M. van Loosdrecht, Merle K. de Kreuk

**Affiliations:** ^1^ Department of Water Management Delft University of Technology Delft The Netherlands; ^2^ Department of Biotechnology Delft University of Technology Delft The Netherlands

**Keywords:** biofilm, diffusion coefficient, error analysis, granular sludge, wastewater treatment

## Abstract

Biofilm and granular sludge processes depend on diffusion of substrates. Despite their importance for the kinetic description of biofilm reactors, biofilm diffusion coefficients reported in literature vary greatly. The aim of this simulation study was to determine to what extent the methods that are used to measure diffusion coefficients contribute to the reported variability. Granular sludge was used as a case study. Six common methods were selected, based on mass balances and microelectrodes. A Monte Carlo simulation was carried out to determine the theoretical precision of each method, considering the uncertainty of various experimental parameters. A model‐based simulation of a diffusion experiment was used to determine the theoretical accuracy as a result of six sources of error: solute sorption, biomass deactivation, mass transfer boundary layer, granule roughness, granule shape, and granule size distribution. Based on the Monte Carlo analysis, the relative standard deviation of the different methods ranged from 5% to 61%. In a theoretical experiment, the six error sources led to an 37% underestimation of the diffusion coefficient. This highlights that diffusion coefficients cannot be determined accurately with existing experimental methods. At the same time, the need for measuring precise diffusion coefficients as input value for biofilm modeling can be questioned, since the output of biofilm models has a limited sensitivity toward the diffusion coefficient.

## Graphical abstract

The authors evaluated six common methods to measure diffusion coefficients of molecules in a biofilm. Virtual experiments and model‐based simulations were used to determine the precision and accuracy of the methods. The methods were imprecise and inaccurate, mainly due to the limited sensitivity of the diffusion coefficient in the diffusion experiments. Thus, diffusion coefficients in biofilms cannot be determined accurately.

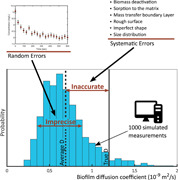

## 1 INTRODUCTION

Many biological wastewater treatment technologies use biofilms to immobilize essential microorganisms. Trickling filters have been used for more than a century to treat wastewater (Daigger & Boltz, 2011) and more recently, anaerobic, aerobic, and Anammox granular sludge have been introduced. In essence, granules are a special form of biofilms, where bacteria are immobilized in auto‐generating biomass particles instead of growing on a carrier surface. Immobilizing the biomass allows high‐rate wastewater treatment because of the efficient separation of granules and treated wastewater. High volumetric conversion rates can be achieved due to the increased liquid/solid mass transfer surface area in granular sludge reactors (Nicolella et al., 2000).

Before a compound can be converted in a biofilm, it has to diffuse into the biofilm. Diffusion has both negative and positive effects on biofilm performance. On the one hand, diffusion will limit the effectiveness of a biofilm. Microorganisms located deeper in the biofilm experience lower substrate concentrations than those located at the biofilm surface. Consequently, the organisms deeper in the biofilm convert substrates at a reduced rate, or are inactive. On the other hand, diffusion creates different redox conditions throughout a biofilm. Therefore, multiple biological reactions can take place within a single reactor (de Kreuk et al., 2005; Vlaeminck et al., 2012) and a separate reactor for each conversion is not required. The overall conversion rates can be steered by controlling the diffusion depth of rate‐limiting soluble substrates (e.g., oxygen, nitrate, ammonium, carbon sources). Therefore, to optimize the conversion rates in biofilm‐based wastewater treatment, a proper understanding of the diffusion process is required.

Extracellular polymeric substances and microbial cells in a biofilm hinder the diffusion of solutes into the biofilm. As a result, the diffusion coefficient for a solute in a biofilm is lower than the diffusion coefficient of the same solute in water (Stewart, 2003). The impact of the biofilm matrix on the diffusion coefficient of a solute depends on the solute properties, which includes size and charge (Hinson & Kocher, 1996; Stewart, 1998), and biofilm properties, such as density (Fan et al., 1990; Horn & Morgenroth, 2006). Many research have studied diffusion of different solutes in different biofilms, with methods such as steady‐state flux measurements (Beyenal & Tanyolac, 1994; Livingston & Chase, 1989; Tang & Fan, 1987; Williamson & McCarty, 1976), transient uptake measurements (Fan et al., 1990; Westrin & Zacchi, 1991), and microelectrode measurements (Fu et al., 1994; Kühl & Jørgensen, 1992; Lewandowski et al., 1991; Revsbech et al., 1986). A review by Stewart (1998) highlighted the wide range of diffusion coefficients described in literature, even for the same solutes. This was partially attributed to differences in biofilm density, but very few studies have been done after publication of this review to verify this hypothesis (Guimerà et al., 2016; Horn & Morgenroth, 2006). The wide range of values makes it difficult to know which diffusion coefficients to use in biofilm models or kinetic analyses. Possibly, as suggested by Stewart (1998), the large variation in diffusion coefficients is the result of the variety of biofilms that exist. Another possibility, that has often been overlooked, is the quality of the methods that were used to determine the biofilm diffusion coefficients. The precision or accuracy in the methods might be an important factor in the reported variation. To our knowledge, the methods to determine biofilm diffusion coefficients have never been reviewed comprehensively. Westrin et al. (1994) have given an overview for diffusion coefficient measurements in hydrogels, but several methods commonly used to study biofilms were not included.

The aim of this paper was to illustrate fundamental shortcomings of methods to measure biofilm diffusion coefficients. To limit the scope of this study, aerobic granular biofilms were used as example case. We selected six common methods and used an uncertainty and sensitivity analysis based on Monte Carlo simulations to determine the theoretical precision of each method. Furthermore, we assessed the theoretical accuracy of one method with simulations of six illustrative examples. The examples were solute sorption, granule deactivation, boundary layer, granule roughness, granule shape, and granule size distribution. We found significant method limitations for both precision and accuracy. Furthermore, we discuss the translation of the results to biofilms in general, as well as the implications of our findings for process engineering of biofilm reactors.

## 2 MATERIALS AND METHODS

### 2.1 Selection of methods

The methods evaluated in this paper were selected based on literature (Stewart, 1998). We chose to exclude light or fluorescence‐based methods as they are generally limited to thin or translucent biofilms. Magnetic resonance based methods are excluded as well, since they only apply to paramagnetic molecules or water. The diaphragm cell was excluded as it does not apply to granular biofilms. Methods 1–3 are based on mass balance calculations, while method 4–6 are based on microelectrode measurements. Note that the steady‐state methods (1, 4, and 5) yield the effective diffusive permeability, while the transient methods (2, 3, and 6) yield the effective diffusivity. If the diffusion process is framed in terms of only the biofilm water volume, the effective diffusivity is the proper parameter. However, if the diffusion process is framed in terms of the whole biofilm volume (including cells and polymeric matrix), the effective diffusive permeability is the right parameter. As a consequence, both parameters typically differ by a factor equal to the porosity. For determination of the steady‐state flux into a biofilm, the effective diffusive permeability is required (see Stewart (1998) for a detailed explanation). The theory of six selected methods is briefly described below, while relevant equations are given in Supporting Information Section 1.2.

**Method 1: Steady‐state reaction**. This method determines the apparent flux of a solute into granules, from the apparent granule area and the concentration change of the solute in the liquid phase. A diffusion‐reaction equation (see Supporting Information Section 1.2) is then solved iteratively, to match the apparent flux into the granules and the liquid phase concentration. The diffusion coefficient is varied to obtain the best fit, thus kinetic constants should be known a priori. This method has been used extensively in the past (Beyenal & Tanyolac, 1994; Livingston & Chase, 1989; Tang & Fan, 1987; Williamson & McCarty, 1976).

**Method 2: Transient uptake of a nonreactive solute**. In this method, granules that are free of solute are placed in a well‐mixed solution of finite volume and known concentration of a solute. The uptake of the solute into the granules follows Fick's 2nd law of diffusion and the diffusion coefficient is obtained by least‐squares fitting of the liquid phase concentration (Crank, 1975, pp. 93–96; Westrin & Zacchi, 1991). This method works with inert molecules or with deactivation of the biomass.

**Method 3: Transient release of a nonreactive solute**. This method is the reverse of the previous method. The granules are soaked with a solute before being placed in a solution of finite volume that is initially free of solute. The increase in liquid phase concentration can be used to obtain the diffusion coefficient (Crank, 1975, pp. 93–96).

**Method 4: Steady‐state concentration profiles inside and outside a granule**. In this method, microelectrodes are used to measure the concentration profile of many small molecules (e.g., oxygen) within a granule. Under steady‐state conditions, the flux into the granule equals the flux through the concentration boundary layer. Both fluxes can be determined from the local concentration gradient and the local diffusion coefficient. If the diffusion coefficient in the boundary layer is known, the diffusion coefficient in the granule can be calculated (Cronenberg & Van Den Heuvel, 1991; Hille et al., 2009; Lewandowski et al., 1991).

**Method 5: Steady‐state reaction with concentration profile inside a granule**. This method is a combination of method 1 and 4 and is useful when the concentration gradient in the boundary layer is not clearly detectable. The apparent flux into a granule can be estimated from the change in liquid phase concentration, granule area and bulk volume (Hille et al., 2009; Horn & Morgenroth, 2006). This apparent external flux equals the internal flux, which can be calculated from the concentration gradient within a granule and the unknown solute diffusion coefficient. When the concentration gradient within a granule is measured with a microelectrode, the diffusion coefficient is the only unknown parameter.

**Method 6: Transient penetration of a solute to the center of a granule**. With a microelectrode tip placed in the center of a single granule and a step‐change in liquid phase concentration, a concentration profile in the center of the granule can be obtained. This profile follows Fick's 2nd law of diffusion and a least‐squares fitting can be used to obtain the diffusion coefficient (Beuling et al., 2000; Crank, 1975, pp. 90–91; Cronenberg & Van Den Heuvel, 1991; Hille et al., 2009).

### 2.2 Model experimental system

To assess precision and accuracy in an easy and flexible manner, virtual experiments were carried out. These virtual experiments were done with a model system: granules with certain properties and a solute with certain properties. For clarity, the properties of the model system were kept constant throughout all simulations (see Table 1). Oxygen was used as the diffusing solute and the reaction kinetics were taken from the first biofilm benchmark problem (Morgenroth et al., 2004). For each of the six methods described in the previous section, an experimental data set was simulated based on the corresponding model equations and experimental parameters (see Table S1). The simulated experimental data set of a method should be similar to a data set that an experimentalist would obtain with that specific method. We chose to simulate experimental datasets instead of using published datasets, to have full control over the input variables and to have a separate evaluation of precision and accuracy. Still, experimental parameters (e.g., experiment duration, microelectrode step size) were taken from literature when possible. A full overview of the experimental parameters, the governing equations, and the resulting simulated experimental data is given in the Supporting Information (Table S1, Equations S1–S8, and Figure S1).

**Table 1:** Characteristics of the granular sludge and solute, which will be used in all subsequent simulations

Parameter	Value	Unit	References
Granule radius (*r*_g_)	1.5e‐3	m	‐
Granule diffusion coefficient (*D*_g_)	1.2e‐9	m^2^/s	Stewart (2003)
Bulk diffusion coefficient (*D*_aq_)	2.0e‐9	m^2^/s	Stewart (2003)
Biomass concentration (*C*_X_)	10,000	gCOD/m^3^	Morgenroth et al. (2004)
Maximum uptake rate (*q*_max_)	3.54	gO_2_/gCOD/d	Morgenroth et al. (2004)
Half saturation coefficient (*K*)	0.2	g/m^3^	Morgenroth et al. (2004)

### 2.3 Simulations to determine precision

The precision of a method refers to the closeness of two or more measured values to each other. Here, the theoretical precision of each method was quantified by the relative standard deviation (RSD) of each method. The RSD was obtained from an uncertainty analysis with Monte Carlo simulations. For each method, typical experimental parameters with corresponding experimental uncertainty were defined. By sampling and propagating this input uncertainty through the measurement methods with Monte Carlo simulations, the theoretical precision of the diffusion coefficient determination could be quantified. The major contributors to the imprecision of the measurements were determined by a sensitivity analysis.

#### 2.3.1 Step 1: Uncertainty analysis

The uncertainty of the parameters that were required as input was estimated based on literature when possible (see Table 2). The uncertainty of the remaining five parameters was estimated to our best knowledge: the total volume (sum of liquid and granule volumes), the liquid phase concentration, and the microelectrode concentration were considered quite well known and a RSD of 1% was chosen. The uncertainty in granule volume and granule radius were set to 5% and 10% respectively, according to our own laboratory experience. Lastly, the granule biomass concentration uncertainty was set to 25%. This high value was deemed reasonable, due to the complexity of estimating the microbial cell concentration in the granule. All parameters were assumed to follow a normal distribution and correlation between parameters was not considered. The parameter space of each method was sampled with Latin Hypercube Sampling (LHS) with 1000 samples (McKay et al., 1979; Sin et al., 2009).

**Table 2:** Parameters used in the Monte Carlo simulations including the RSD involved in each parameter and the method, described in Section 2.1, they are required for

Parameter	RSD (%)	Methods	References
Granule volume (*V*_G_)	5	1–3, 5	‐
Total volume (*V*_T_)	1	1–3, 5	‐
Granule radius (*r_g_*)	10	1–3, 5, 6	‐
Bulk concentration (*C*_B_)	1	1–3, 5, 6	‐
Biomass concentration (*C*_X_)	25	1	‐
Half saturation constant (*K*)	50	1	Sin et al. (2009)
Maximum uptake rate (*q*_max_)	5	1	Sin et al. (2009)
Microelectrode concentration (*C*_M_)	1	4, 5, 6	Bryant et al. (2010)
Microelectrode step size (dx)	10	4, 5	Cronenberg and Van Den Heuvel (1991)

*Note*: The uncertainty was approximated to be either 1%, 5%, 10%, 25%, or 50% RSD, since more accurate estimates could not be made.

Abbreviation: RSD, relative standard deviation.

#### 2.3.2 Step 2: Model simulation

The Monte Carlo simulations were carried out for each of the six methods (Section 2.1) separately. For each method, 1000 LHS‐sampled datasets were used to fit the simulated experimental datasets of step 1. The procedure to fit the datasets is given in Supporting Information Section 1.2. Due to the changing input parameters, each Monte Carlo simulation step resulted in a slightly different diffusion coefficient. The 1000 combined diffusion coefficients yielded a distribution with a certain standard deviation. The distribution was checked visually for normality, and the RSD was used as the precision of the method. The difference in the diffusion coefficient used to simulate the experimental data set (Table 1) and the mean of the diffusion coefficient distribution, was used as a measure of the inherent accuracy of the method.

#### 2.3.3 Step 3: Sensitivity analysis

A sensitivity analysis was performed to determine the relative importance of the input parameters in the uncertainty in the diffusion coefficient. The analysis consisted of a multivariate linear regression of the model output (diffusion coefficient) on the model inputs (Saltelli et al., 2008). The standardized regression coefficients, *β*_i_, were obtained by mean‐centered sigma‐scaling (Helton & Davis, 2003). The model was considered sufficiently linear if the coefficient of determination (*R* ^2^) was equal to or larger than 0.7 (Sin et al., 2011). An input parameter was considered significant only if its absolute *β*_i_^2^ value was >0.01 (Sin et al., 2011). For a concentration profile in time or space, each data point gave a unique regression coefficient. In that case, the *β*_i_^2^ values of each data point were summed together to obtain a *β*_i_^2^ that represents the aggregate impact of the uncertainty in concentration measurements.

### 2.4 Simulations to determine accuracy

Accuracy refers to how close a measured value is to a true value. The accuracy of a method can be limited by simplifications of real conditions, which are often needed to estimate diffusion coefficients in granules. The simplifications that lead to inaccurate measurements are also called systematic errors. The impact of such systematic errors is assessed in this paper. We have selected several potential errors based on prevalence and potential impact, according to our own insight. The potential errors are meant to be illustrative and therefore do not necessarily apply to all the methods described in Section 2.1. The estimation of the inaccuracy due to solute sorption and biomass deactivation, was based on Stewart (1996) and Stewart (1998), respectively. A detailed description is given in Supporting Information Section 1.4.

**Figure 1:**
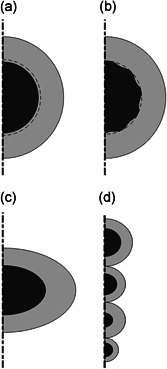
Geometry of the four systematic errors that were simulated in COMSOL, based on the typical values. The black area represents the granule, the gray area represents the bulk liquid, and the dash‐dotted line represents the axis of symmetry. (a) Concentration boundary layer, with the dashed line indicating the layer thickness. (b) Surface roughness, with the dashed line indicating the liquid volume within the maximum granule diameter where no convection occurs. (c) Granule shape. (d) Granule size distribution, with one bigger, one smaller, and two average granules. Note that figure (d) is not drawn to scale

The impact of several other errors is estimated with a mathematical model, which compares the experiment with and without the assumptions. The model is based on method *transient uptake of a nonreactive solute*, as described in Section 2.1. The granule is simulated with a 2D‐axisymmetric model. The base model consisted of a single granule in water with an α (the ratio of liquid volume over granule volume) of 4. The initial solute concentration in the liquid was 10 g/L, while the granule was initially free of solute. Other granule characteristics are as described in Table 1. The diffusion coefficient in the bulk liquid was set to an artificially high value of 1 × 10^9^ m^2^/s to simulate a perfectly mixed reservoir. The model simulated the concentration change over time until equilibrium was reached. The concentration data of the bulk liquid were extracted from COMSOL and used as input data to determine the diffusion coefficient (according to the standard procedure for method *transient uptake of a nonreactive solute*, as described in Section 2.1). The standard procedure did not consider any systematic error and the difference between the diffusion coefficient that was used in COMSOL (1.2 × 10^−9^ m^2^/s) and the fitted diffusion coefficient therefore equalled the inaccuracy caused by the simplifications of the measurement. The following systematic errors were considered in COMSOL (see Figure 1):

- **Mass transfer boundary layer (MTBL):** A MTBL was added to the model. The MTBL thickness was set to 100 µm (estimated based on Horn & Morgenroth, 2006; Rasmussen & Lewandowski, 1998), with a diffusion coefficient of 2 × 10^−9^ m^2^/s.
- **Surface roughness:** The granule surface in the model was changed from smooth to sinusoidal. The amplitude of the sine wave was 50 µm and the period was set to 10 sine waves for the full granule radius (see Figure 1b). The average granule radius was kept at 1.5 mm, and therefore the granule volume remained unchanged. The diffusion coefficient in the pores (liquid volume within the maximum granule diameter) was set to 2 × 10^−9^ m^2^/s.
- **Granule shape:** The shape of a granule was changed to an oblate spheroid, with a length of its semimajor axis of 1.80 mm and a length of its semiminor axis of 1.04 mm. The spheroid had an equivalent spherical diameter of 1.5 mm and a sphericity of 0.95.
- **Granule size distribution:** The model was extended to four differently sized granules to simulate the spread of granule radii present in a sample (Westrin & Zacchi, 1991). Two granules had a radius of 1.5 mm, one granule had a smaller radius of (1.5 − *δ*) mm, and the last granule had a larger radius of (1.5 + δ) mm. Here, *δ* is the deviation from the mean diameter. It was set to 0.5 mm.

A sensitivity analysis was carried out for each systematic error to investigate the influence of the chosen parameters on the accuracy. The values described here were used as typical values.

## 3 RESULTS AND DISCUSSION

### 3.1 Precision

**Figure 2:**
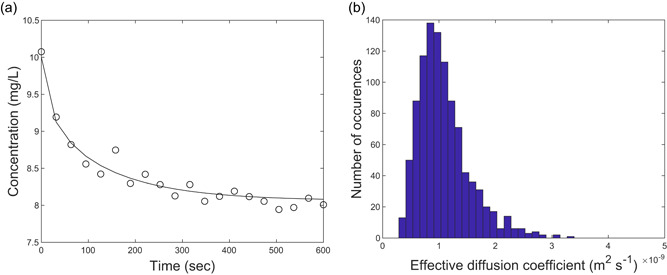
Uncertainty analysis output for the *transient uptake of a nonreactive solute* method. (a) Simulated data set with simulated experimental data (dots) and example model fit (solid line). (b) Distribution of fitted diffusion coefficient for 1000 Monte Carlo simulations [Color figure can be viewed at wileyonlinelibrary.com]

A Monte Carlo uncertainty analysis was used to determine the theoretical precision of six common methods to estimate diffusion coefficients. The analysis yielded the precision and the inherent accuracy of each method. The precision was defined as the RSD. The inherent accuracy was defined as the difference between the true diffusion coefficient used to simulate the experimental datasets and the average fitted diffusion coefficient. An example of the output for one method (*transient uptake of a nonreactive solute*) is given in Figure 2. The uncertainty analysis to quantify the precision of each method revealed a wide spread among the methods (see Table 3). The RSD ranges from 5% (*steady‐state concentration profiles inside and outside a granule*) to 61% (*steady‐state reaction*). This wide range shows that there are significant differences between the methods. It suggests that the impreciseness of the methods could indeed be a major source of the wide range of diffusion coefficients reported in literature (Stewart, 1998). Strikingly, the *steady‐state reaction* method is simultaneously the least precise method and one of the most frequently used methods in past research (Arvin & Kristensen, 1982; Beyenal & Tanyolac, 1994; Herrling et al., 2015; Khlebnikov et al., 1998; Livingston & Chase, 1989; Mulcahy et al., 1981; Tang & Fan, 1987; Wagner & Hempel, 1988; Wang & Tien, 1984; Williamson & McCarty, 1976; Yano et al., 1961; Yu & Pinder, 1994).

**Table 3:** Results of Monte Carlo simulation with RSD and inherent inaccuracy per method

Method		RSD (%)	Inaccuracy (%)
**1**	Steady‐state reaction	61	19
**2**	Transient uptake of a nonreactive solute	42	−10
**3**	Transient release of a nonreactive solute	33	5
**4**	Steady‐state concentration profiles inside and outside a granule	5	19
**5**	Steady‐state reaction with concentration profile inside a granule	12	16
**6**	Transient penetration of a solute to the center of a granule	20	−1

*Note*: A complete overview of the sensitivity analysis results is given in Table S3.

Abbreviation: RSD, relative standard deviation.

#### 3.1.1 Mass balance based methods

None of the mass balance based methods (methods 1–3) were precise, with RSD always greater than 33%. A clear comparison between our simulated precision and experimental precisions reported in literature was not possible. Diffusion experiments in a laboratory are often carried out only once, due to the time and effort required per experiment. This also limits the usefulness of replicate measurements. The standard error of the mean of an experiment is given by $\sigma_{\overset{®}{x}}=\frac{\sigma }{\sqrt{n}}$, where σ is the *SD* of the method and *n* is the number of replicates. With a RSD of 33%, the relative standard error is 19%, 15%, or 10% for 3, 5, and 10 replicate measurements, respectively.

The uncertainty in the granule volume was a major source of imprecision for the *transient uptake and transient release of a nonreactive solute methods*. The input uncertainty was only 5%, but it accounted for 31% and 57% of the total uncertainty of the *transient uptake* and *transient release* methods, respectively (see Table S3). The *transient release* and *transient uptake* methods are quite similar, but the *release* method is more precise. This could be expected, since the relative concentration change in the *release* method is greater than in the *uptake* method (see Figure S1). As a result, the concentration uncertainty accounted for <1% and 33% of the total uncertainty of the *transient release* and *transient uptake* methods, respectively. For the *steady‐state reaction* method, the biomass concentration in the granule was the major source of uncertainty (49% of total uncertainty). This parameter is not easily measured, but it apparently plays a significant role for this method. The granule radius also had a major effect on the precision of the methods (6–37% of total uncertainty).

A substantial inherent inaccuracy was present for the *steady‐state reaction* method (19%), which could be caused by nonlinearity of the data processing. The distribution of diffusion coefficients of the Monte Carlo analysis is skewed, indicating that input uncertainties are amplified more in one direction than the other (see Figure S2). This inherent inaccuracy is difficult, if not impossible, to identify with conventional experiments. In our analysis, the inherent inaccuracy in the data processing could be identified, because we used virtual experiments. The diffusion coefficient used to design these virtual experiments was known and could directly be compared with the output diffusion coefficient. The inherent inaccuracy of the other methods can be found in Table 3.

#### 3.1.2 Microelectrode based methods

Overall, microelectrode based methods (methods 4–6) were more precise than mass balance based methods. The RSD was always 20% or lower, which agrees with Etterer (2006). However, Chiu et al. (2007) report a higher RSD of 30–40%. Microelectrode measurements are highly localized and rely less on granule parameters, like granule volume, granule area, or granule radius. They are thus less impacted by the relatively large uncertainty in these parameters (see Table 2). For microelectrodes, many replicates are done relatively easy (Horn & Morgenroth, 2006). However, these replicates cannot be considered as true repeat measurements. The granules are heterogeneous and multiple granules may have different diffusive properties (Wilén et al., 2004). The repeat measurements in microelectrode studies are required to average spatial heterogeneity. Van Loosdrecht et al. (1995) and Ning et al. (2014) have shown that oxygen profiles at multiple locations can differ significantly and lead to a wide range of calculated flux values.

Similar to the mass balance based methods, the largest sources of uncertainty for the microelectrode based methods are the granule volume and granule radius (see Table S3). Input parameters that are specifically related to microelectrodes are of limited importance. Only for the *steady‐state concentration profiles inside and outside a granule* method is the relative impact of microelectrode concentration measurement and microelectrode position significant. However, given the overall high precision of this method (5% RSD), their absolute impact is small. The *steady‐state reaction with concentration profile inside a granule* is affected by both the granule radius (74% of total uncertainty) and the granule volume (19% of total uncertainty). The dependence on these uncertain parameters leads to a total uncertainty of 12% RSD. Still, it is significantly more precise than the mass balance based methods. The *transient penetration of a solute to the center of a granule* is almost solely affected by the granule radius, resulting in a lower precision as well (compared to *steady‐state concentration profiles inside and outside a granule*).

#### 3.1.3 Recommended method for granular sludge

The ideal method to measure diffusion coefficients can be used for a wide range of solutes, measures diffusion coefficients on a global scale, and combines a high precision with a high accuracy. It is clear that none of the methods analysed meet these requirements. Microelectrodes offer high precision, but they are limited to the molecules for which a microelectrode is readily available and they only measure locally. We therefore recommend the use of the second method, *transient uptake of a nonreactive solute*, for future experiments. Since its large imprecision makes this method far from ideal, it is an inferior method that is nonetheless the best option. Despite its large imprecision, this method has the potential to determine the order of magnitude of diffusion coefficients for a wide range of solutes. The third method, *transient release of a nonreactive solute*, is more precise, but we expect more practical issues. For example, one major issue is transferring granules soaked with some solute from one solution to another, without transferring excess water that is retained between granules. Note that this recommendation is specific for spherical biofilms, for flat biofilms a diaphragm cell might be the preferred option (see Section 3.4).

### 3.2 Accuracy

**Figure 3:**
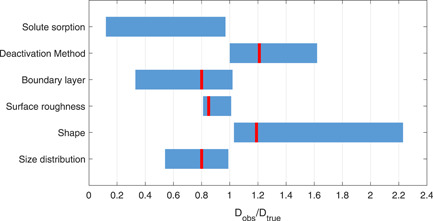
Simulated effect of different systematic errors on the observed diffusion coefficient. The bars represent the range of errors that result from the sensitivity analysis (see Figure S3–S8). The red lines indicate the inaccuracy for the typical case [Color figure can be viewed at wileyonlinelibrary.com]

The impact of six systematic errors was estimated with analytical calculations (solute sorption and deactivation method) and with a COMSOL model (mass transferboundary layer, surface roughness, shape, and size distribution). The impact of the different systematic errors on the observed diffusion coefficient is shown in Figure 3. The figure displays a wide range of under‐ and overestimations of the true diffusion coefficient. In the most extreme case, the observed diffusion coefficient is more than twice as high as the true diffusion coefficient.

#### 3.2.1 Sorption

Binding of solutes to the granule matrix creates an underestimation of the true diffusion coefficient. A solute that enters a granule has to distribute according to the concentration gradient. If part of the solute binds to the granule matrix, more solute needs to enter the granule before equilibrium is reached. This will require more time and thus lead to an underestimation of the diffusion coefficient. This error only plays a role with transient methods, since in steady‐state the binding of solutes to the matrix is in equilibrium. The nature of the solute will often reveal whether adsorption will be a problem. Hydrophobic molecules (e.g., phenols, phthalates) or charged molecules (e.g., ammonium) are much more likely to adsorb than hydrophilic, neutral molecules. Even though there are some reports that indicate oxygen can adsorb to bacterial cell walls (Beuling et al., 2000; Möller et al., 2005), it is unclear how significant this effect would be for a biofilm. Therefore, no typical error is included.

#### 3.2.2 Deactivation

Permeabilisation of microbial cells leads to an increase in the area available for diffusion, and thereby to a significant overestimation of the diffusion coefficient (up to 60%). Some molecules, such as oxygen, can already diffuse through the cells and will therefore be less impacted (Beuling et al., 2000). Experimental work with nuclear magnetic resonance by Lens et al. (2003) and microelectrodes by Lens et al. (1993) revealed an inaccuracy of similar magnitude due to deactivation of methanogenic granules. Deactivation with glutaraldehyde and mercuric chloride were notable exceptions. Glutaraldehyde caused an underestimation, most likely because it does not permeabilise cells (Azeredo et al., 2003) and even forms cross‐links in the EPS matrix (McDonnell & Russell, 1999). Mercuric chloride did not lead to an inaccuracy, but literature reports on its effect are conflicting (Ames et al., 1986; Fu et al., 1994; Matson & Characklis, 1976; Valko & DuBois, 1944).

#### 3.2.3 Mass transfer boundary layer

Negligence of the MTBL can result in a clear underestimation of the true diffusion coefficient. The layer provides an additional resistance for the diffusing solute, and thus the concentration change will be slower. The error increases with increasing boundary layer thickness. The thickness values tested ranged from 0 to 800 μm, with 100 μm as a typical value (estimated based on Horn & Morgenroth, 2006; Rasmussen & Lewandowski, 1998). Reducing the thickness of the boundary layer is not trivial, since it depends on the liquid properties, as well as the slip velocity of the granules (Van Benthum et al., 1999).

#### 3.2.4 Surface roughness

A rough granule surface resulted in a small underestimation of the diffusion coefficient. This may seem counterintuitive, since the surface area of a granule increases with its roughness. A higher surface area should lead to an overestimation. However, in the simulation, mass transfer in the liquid volume in the granule valleys was through diffusion only (Picioreanu et al., 2000). The total distance a solute has to diffuse increases with surface roughness, and thus the diffusion coefficient is underestimated. This is in accordance with the findings of Picioreanu et al. (2000), who found that smooth biofilm surfaces allow for maximum mass transfer. Overall, the impact of this error is small, since the roughness amplitude (≤100 μm) is small compared to the granule radius (1500 μm).

#### 3.2.5 Shape

Negligence of granule shape can cause a significant overestimation of the diffusion coefficient (up to 120%). A spheroidal granule has a larger surface‐to‐volume ratio than a perfectly spherical granule. The increase in area leads to a faster change in liquid concentration and thus an overestimation of the diffusion coefficient. Since literature reports of spheroidal granules are common, this is an error that might play a large role (Csikor et al., 1994; Gjaltema et al., 1995; Li et al., 2013; Liu et al., 2006; Schmidt & Ahring, 1996). A correction factor to the measured diffusion coefficient based on observed granule shape might solve this problem partially.

#### 3.2.6 Size distribution

A size distribution of the granular sludge sample can introduce a moderate underestimation of the diffusion coefficient. In the simulation of size distribution, both a smaller granule fraction and a bigger granule fraction are included. Diffusion into the smaller granules proceeds much faster, while diffusion into the larger fraction is much slower. The combined effect is not readily predicted, but the simulation reveals that the larger fraction has a bigger effect. The smaller fraction only impacts the initial concentration change, while the larger fraction increases the time required to reach equilibrium. Therefore, the larger fraction impacts the whole concentration profile, while the smaller fraction only affects the initial part. Our results match with those found by Westrin and Zacchi (1991), who used a similar method to test the impact of the size distribution.

#### 3.2.7 Combined effect

The exact effect of the systematic errors is difficult to quantify, since multiple systematic errors might cancel out. However, it seems just as reasonable to expect additive effects of different errors. After all, four out of the six simulated errors lead to underestimations of the diffusion coefficient. If we assume that all errors are multiplicative, we obtain an underestimation of 37% (*D*_observed_/*D*_true_ = 0.63). This highlights the importance of the systematic errors and the need for a thorough analysis of the assumptions that are made. Obviously, other errors, that are not part of this study, can play a role as well. Still, the results highlight that the overall effect can be substantial. We recommend experimentalists to routinely check their diffusion methods for systematic errors to maximize accuracy.

### 3.3 Sensitivity of the diffusion coefficient

At first glance, the simulations of precision and accuracy suggest that the granule surface area is the core parameter that limits the methods. The granule surface area was not included explicitly in the Monte Carlo simulations, but it was implicitly derived from the granule volume and radius. Exactly these two parameters were the biggest contributors to the imprecision of the methods. Furthermore, three out of the six simulated systematic errors (surface roughness, shape, and size distribution) are related to surface area. However, the impact of granule surface area cannot explain all simulation results. For example, the simulation results for method 5 (*Steady‐state reaction with concentration profile inside a granule*) suggest that the impact of the granule surface area is limited. Method 5 depends on granule volume and radius, but it is still quite accurate (12% RSD).

We believe that there is another, more significant reason that limits precision and accuracy. The two most precise methods (methods 4 and 5, see Table 3) are based on a direction evaluation of Fick's 1st law. The measurement of the concentration gradient with microelectrodes and a direct measurement of flux allow to directly estimate the diffusion coefficient. In contrast, the four least accurate methods (methods 1, 2, 3, and 6) are based on derivations of Fick's 2nd law. This difference might seem trivial, but that is not the case. We found that all input uncertainties were amplified in methods that depend on Fick's 2nd law. For example, the uncertainty in the granule radius was set to 10% (see Table 2). If we carry out a Monte Carlo simulation without considering the uncertainty in other parameters, the precision of method 1, 2, 3, and 6 was always 20% RSD. For other parameters (e.g., granule volume, concentration) the result were amplified by a factor of two as well. This amplification was not observed in methods 4 and 5, which are based on Fick's 1st law. For method 5, a 10% uncertainty in the granule radius led to a precision of 10% RSD.

The aforementioned amplification of uncertainty in Fick's 2nd law suggests that the diffusion coefficient is a parameter with limited sensitivity. Any uncertainty in sensitive parameters (granule volume, radius, etc.) is amplified, leading to imprecise estimates of the diffusion coefficient. This amplification can explain why for some methods, the precision is much worse than the uncertainty of input parameters. For example, method 3 has input uncertainties of 1%, 5%, and 10%, but the method precision is 33%. Other authors have also found a reduced or limited sensitivity of the diffusion coefficient, at least under certain conditions (Boltz et al., 2011; Harremoës, 1978; Harris & Hansford, 1976; Morgenroth et al., 2004). This reduced sensitivity of the diffusion coefficient is the core reason why diffusion coefficients cannot be measured accurately. Only methods based on Fick's 1st law do not suffer from the reduced sensitivity, but those methods require measurements of the concentration gradient. This means that these methods measure locally and are limited to solutes for which localized measurements are possible.

### 3.4 Translation to other biofilm types

Even though granular biofilms are an important application of biofilms in wastewater treatment, more biofilm types are being used. Other processes that rely on biofilms are the tricking filter, the moving bed biofilm reactor, the membrane biofilm reactor, and the rotating biological contactor. We believe that the two major reasons that limit diffusion experiments for granular biofilms (biofilm surface area and diffusion sensitivity) apply to flat biofilms as well.

The surface area of biofilm carriers is well‐defined, but the actual biofilm surface area is more difficult to estimate. Biofilms growing on carriers can have rough surfaces and the thickness can be nonuniform (e.g., see the figures in Gapes & Keller, 2009; Ødegaard, 2006). Furthermore, the relation between biofilm surface area and biofilm volume is not per definition constant with biofilm thickness. Many biofilm carriers have irregular geometries and assuming flat geometry can introduce a systematic error for thicker biofilms. The geometry of typical carriers might also lead to imperfect mixing and MTBLs within the carrier (Gapes & Keller, 2009; Nogueira et al., 2015; Tang et al., 2017). Even if the estimate of biofilm surface area would be more precise, this does not mean that the methods are more precise. The methods based on Fick's 2nd law still amplify the input uncertainty. Fick's 2nd law can be used to describe both flat and spherical geometry, although the formulation will be slightly different. Thus, the reduced sensitivity of the diffusion coefficient applies to flat biofilms as well.

For flat biofilms, the diaphragm cell is also frequently used to measure biofilm diffusion coefficients (Horn & Morgenroth, 2006). A preliminary Monte Carlo simulation of this method showed that precise results can be achieved, with a RSD of 5% (data not shown). This implies that diffusion coefficients can be measured more precisely in flat biofilms than in spherical ones. However, biofilms have to be either grown directly on the diaphragm membrane or they have to be transferred from their natural environment onto the membrane. The biofilms have to be the exact same shape and size as the membrane to prevent leakage of solutes around the biofilm. For example, Bryers and Drummond (1998) have shown that channels in a biofilm can lead to clear overestimation of the diffusion coefficient. Therefore, although a diaphragm cell is precise, we expect that the measured diffusion coefficients are still relatively inaccurate. Obviously, experimental verification of this hypothesis is required.

Overall, we expect the findings of this paper to translate quite well to other biofilm types. We recommend researchers who want to measure diffusion coefficients in flat biofilms to perform a similar analysis to verify the precision and accuracy of their method of choice.

### 3.5 Implications for biofilm modeling

Biofilm models are commonly used to predict performance and improve understanding of biofilm reactors. These models often rely on diffusion coefficients, which raises the question how these models are impacted by our findings. At first, it might seem likely that the descriptive and predictive power of the models is reduced with less accurate diffusion coefficients. However, we expect the impact to be limited. The principles that apply to diffusion experiments apply to biofilms models as well. Namely, biofilm models also require input parameters (biofilm thickness, surface area, etc.) that are measured with a certain precision. Furthermore, simplifications that lead to inaccuracy are often implemented in biofilm models as well (Boltz et al., 2010).

The most important reason why inaccurate diffusion coefficients have a limited impact on biofilm models is the sensitivity of the diffusion coefficient toward the predicted flux of solutes into the biofilm. We showed that the diffusion coefficient is a parameter with limited sensitivity in methods based on Fick's 2nd law. Biofilm models typically employ this same law (together with a reaction term) to determine the flux of a solute in or out of a biofilm. It is this flux combined with the biofilm area that ultimately determines the changes in bulk liquid concentration. It has been shown previously that the flux for zero‐order kinetics is roughly proportional to the square root of the diffusion coefficient (Harremoës, 1978; Harris & Hansford, 1976). We briefly tested this relationship for Monod kinetics, with a numerical diffusion‐reaction model, a single rate‐limiting substrate, constant concentration at the granule surface, and parameters from Table 1 (see Supporting Information Section 2.5). We observed that a 10% change in the diffusion coefficient led to a change in the flux between 0% and 6% (depending on the penetration depth, see Supporting Information Section 2.5 for full results). Obviously, these preliminary results should be rigorously verified in future research to determine if there are certain conditions under which the reduced sensitivity does not apply.

**Figure 4:**
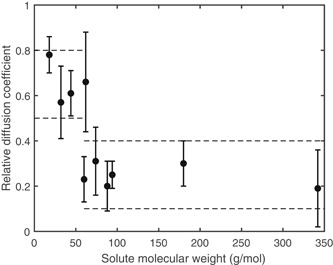
Relative diffusion coefficients (*D*_biofilm_/*D*_aq_) of different solutes in biofilms, redrawn based on Stewart (2003). The dashed lines indicate the recommended approach with relative diffusion coefficients of 0.5–0.8 and 0.1–0.4. Note that the error bars represent the *SD* of the values reported in literature. They do not represent the experimental accuracy. For precise values, see Table S4

Interestingly, the exact reasons why diffusion coefficients cannot be measured with accuracy are simultaneously the reasons why accurate values are not required. Therefore, a better, more accurate method will only marginally improve biofilm models. We suggest to treat biofilm diffusion coefficients as imprecise parameters. Practically, this means that biofilm models do not require a unique diffusion coefficient for each solute. A quick analysis of literature values of common solutes reveals that a high (0.5–0.8) and a low (0.1–0.4) relative diffusion coefficient might be sufficient (see Figure 4), given the accuracy of the diffusion coefficients. Future research could classify molecules into the fast diffusion group and slow diffusion group, as well as determine the approximate diffusion coefficient of larger molecules.

## 4 CONCLUSION

In this simulation study, the theoretical precision of six different methods to measure biofilm diffusion coefficients was evaluated, as well as the theoretical accuracy for one of those methods. The precision of all methods was affected by uncertainty in experimental parameters, although the extent differed per method (RSDs of 5–61%). The precision of microelectrode based methods was higher than that of mass balance based methods. The least precise method, *steady‐state reaction*, has often been used in past research. The experimental parameters with the biggest impact were granule volume, granule radius, and biomass concentration in the granule. These parameters are difficult to identify experimentally and a direct solution for more precise measurements could not be identified. The inaccuracy of the *mass balance–uptake* method was significant, which reduces the reliability of the diffusion coefficient measurements even further. The exact impact of the systematic errors could not be quantified, but an underestimation of the true diffusion coefficient by more than 30% is likely.

Accurate methods for diffusion coefficient measurements are currently not available, but from the point of view of biofilm kinetics they are also not required. The limitations of diffusion coefficient measurements (uncertain experimental parameters, process simplifications, and reduced sensitivity to the diffusion coefficient) apply to biofilm models as well. An imprecise diffusion coefficient will most likely not have a big impact on the descriptive and predictive performance of biofilm models. It might be sufficient to use two relative diffusion coefficients in biofilm models: a high value of 0.5–0.8 for small solutes, such as oxygen, and a low value of 0.1–0.4 for medium‐sized solutes, such as glucose and acetate.

## AUTHOR CONTRIBUTIONS

Lenno van den Berg planned and carried out the simulations. All authors contributed to the interpretation of the results. Lenno van den Berg took the lead in writing he manuscript. Mark C. M. van Loosdrecht and Merle K. de Kreuk provided critical feedback and helped shape the research, analysis and manuscript.

## Supporting information

Supporting information
