# Supplementary material for: How to measure diffusion coefficients in biofilms: A critical analysis
Source: Biotechnol Bioeng. 2020 Dec 25;118(3):1273–85. doi: 10.1002/bit.27650 (PMC7986928; doi:10.1002/bit.27650)
Supplement: Supplementary file 1 — Supporting information [file BIT-118-1273-s001.docx]

**Supplementary information**

**How to Measure Diffusion Coefficients in Biofilms: A Critical Analysis**

Lenno van den Berg^1*^, Mark C.M. van Loosdrecht^2^, Merle K. de Kreuk^1^

^1^ Department of Water Management, Delft University of Technology, The Netherlands

^2^ Department of Biotechnology, Delft University of Technology, The Netherlands

*Corresponding author. Stevinweg 1, 2628 CN Delft, The Netherlands. E-mail: [L.vandenBerg@tudelft.nl](mailto:L.vandenBerg@tudelft.nl)

1. **Materials & Methods**
   1. **Input parameters for random error analysis**

The input parameters for the different methods are given below. This includes the parameters required to simulate the data and the parameter required to fit the experimental data. The parameters that are required to fit the experimental data are all uncertain, with the uncertainty listed in the main paper.

**Table S1. Overview of input parameters for each method that is included in the random error analysis.**

| **Method** | **Parameter** |  | **Unit** | **Value** |
| --- | --- | --- | --- | --- |
| 1 | Total volume | V | m^3^ | 150e-6 |
| 1 | Granule volume | V_G_ | m^3^ | 30e-6 |
| 1 | Bulk concentration | C_B_ | g/m^3^ | 10 |
|  |  |  |  |  |
| 2 | Total volume | V_T_ | m^3^ | 150e-6 |
| 2 | Granule volume | V_G_ | m^3^ | 30e-6 |
| 2 | Initial bulk/granule concentration | C_B_ | g/m^3^ | 10 |
| 2 | Experiment duration | t_range_ | min | 10 |
| 2 | Concentration measurements | n | - | 20 |
|  |  |  |  |  |
| 3 | Total volume | V_T_ | m^3^ | 150e-6 |
| 3 | Granule volume | V_G_ | m^3^ | 30e-6 |
| 3 | Initial bulk/granule concentration | C_B_ | g/m^3^ | 10 |
| 3 | Experiment duration | t_range_ | min | 10 |
| 3 | Concentration measurements | n | - | 20 |
|  |  |  |  |  |
| 4 | Bulk concentration | C_B_ | g/m^3^ | 10 |
| 4 | Microelectrode step size | rx | m | 10e-6 |
| 4 | Microelectrode penetration depth | d_micro_ | m | 300e-6 |
| 4 | Boundary layer thickness | r_CBL_ | m | 100e-6 |
|  |  |  |  |  |
| 5 | Total volume | V_T_ | m^3^ | 150e-6 |
| 5 | Granule volume | V_G_ | m^3^ | 30e-6 |
| 5 | Bulk concentration | C_B_ | g/m^3^ | 10 |
| 5 | Microelectrode step size | rx | m | 10e-6 |
| 5 | Microelectrode penetration depth | d_micro_ | m | 300e-6 |
| 5 | Boundary layer thickness | r_CBL_ | m | 100e-6 |
|  |  |  |  |  |
| 6 | Initial bulk concentration | C_B_ | g/m^3^ | 10 |
| 6 | Experiment duration | t_range_ | min | 15 |
| 6 | Measurement interval | t_meas_ | sec | 20 |

- 1. **Model equations for random error analysis**
     1. **Steady-state reaction**

The flux into the granule (*J* in g/m^2^/s) is calculated based on the bulk volume (*V_Bulk_ in m^3^*), the bulk concentration change over time ((*dC/dt)_Bulk_* in g/m^3^/s), and the granule area (*A_Granule_* in m^2^):

| $V_{Bulk}\left( \frac{dC}{dt} \right)_{Bulk}=J\cdot A_{Granule}$ | (S1) |
| --- | --- |

The diffusion-reaction equation is then solved iteratively, to match the flux into the granule and the bulk concentration. The diffusion coefficient is varied to obtain the best fit. If the substrate uptake rate follows Monod kinetics, the diffusion-reaction equation is defined as follows:

| $D_{Granule}\left( \frac{\partial^{2}C}{\partial r^{2}}+\frac{2}{r}\frac{\partial C}{\partial r} \right)=q_{max}\frac{C}{K_{S}+C}C_{X},$ | (S2) |
| --- | --- |

where *D_Granule_* is the diffusion coefficient (m^2^/s), *r* is the radial position in the granule (m), *q_max_* is the maximum uptake rate (1/s), *C* is the solute concentration within the granule (g/m^3^), *K_S_* the half-saturation coefficient (g/m^3^), and *C_X_* the biomass concentration (g/m^3^).

- - 1. **Transient uptake of a non-reactive solute**

The equation that relates the concentration profile to the diffusion coefficient was derived by Crank (1975, pp. 93-96):

| $\frac{C_{Bulk}\left( t \right)}{C_{Bulk}\left( 0 \right)}=\frac{1}{1+\alpha}\left( \alpha+\sum_{n=1}^{\infty} \frac{6\alpha\left( 1+\alpha\right)\exp-\frac{D_{Granule}q_{n}^{2}t}{r_{Granule}^{2}}}{9+9\alpha+q_{n}^{2}\alpha^{2}} \right),$ | (S3) |
| --- | --- |

where *C_Bulk_(t)* is the bulk liquid solute concentration at time *t* (g/m^3^), *α* is the ratio of bulk volume over granule volume, *r_Granule_* is the granule radius (m), and the *q_n_* values are the non-zero positive roots of the following non-linear equation:

| $\tan q_{i}=\frac{3q_{i}}{3+\alpha q_{i}^{2}}$ | (S4) |
| --- | --- |

- - 1. **Transient release of a non-reactive solute**

The method can also be applied to monitor the release of a solute from the granule into the bulk liquid (Crank, 1975, pp. 93-96).

| $\frac{C_{Bulk}\left( t \right)}{C_{Granule}\left( 0 \right)}=\frac{1}{1+\alpha}\left( 1-\sum_{n=1}^{\infty} \frac{6\alpha\left( 1+\alpha\right)\exp-\frac{D_{Granule}q_{n}^{2}t}{r_{Granule}^{2}}}{9+9\alpha+q_{n}^{2}\alpha^{2}} \right)$ | (S5) |
| --- | --- |

Here, α and q_n_ are defined as in the previous method, while C_Granule_(0) is the initial concentration inside the granules.

- - 1. **Steady-state concentration profiles inside and outside a granule**

The diffusion coefficient in the granule can be calculated from the diffusion coefficient in water (*D_aq_* in m^2^/s), the concentration gradient in the boundary layer ((*dC/dr)_BoundaryLayer_* in g/m^4^), and the concentration gradient in the granule (*(dC/dr)_Granule_* in g/m^4^) (Cronenberg & Van Den Heuvel, 1991; Lewandowski, Walser, & Characklis, 1991):

| $D_{Granule}=D_{aq}\frac{\left( \frac{dc}{dr} \right)_{Boundary Layer}}{\left( \frac{dc}{dr} \right)_{Granule}}$ | (S6) |
| --- | --- |

- - 1. **Steady-state reaction with concentration profile inside a granule**

The diffusion coefficient can be calculated from the flux into the granule and the concentration gradient in the granule. The flux into the granule is determined from the bulk volume, the biofilm area and the bulk concentration change (Horn & Morgenroth, 2006):

| $D_{Granule}=\frac{J}{\left( \frac{dc}{dr} \right)_{Granule}}=\left( \frac{dc}{dt} \right)_{Bulk}\cdot\frac{V_{Bulk}}{A_{Granule}}\cdot\frac{1}{\left( \frac{dc}{dr} \right)_{Granule}}$ | (S7) |
| --- | --- |

- - 1. **Transient penetration of a solute to the centre of a granule**

The profile can be calculated with the following equation (Crank, 1975, pp. 90-91):

| $\frac{C_{Granule}(t)}{C_{Bulk}}=1+2\sum_{n=1}^{\infty} \left( -1 \right)^{n}\cdot\exp-\frac{D_{Granule}n^{2}\pi^{2}t}{r_{Granule}^{2}}$ | (S8) |
| --- | --- |

- 1. **Resulting simulated datasets for random error analysis**

| 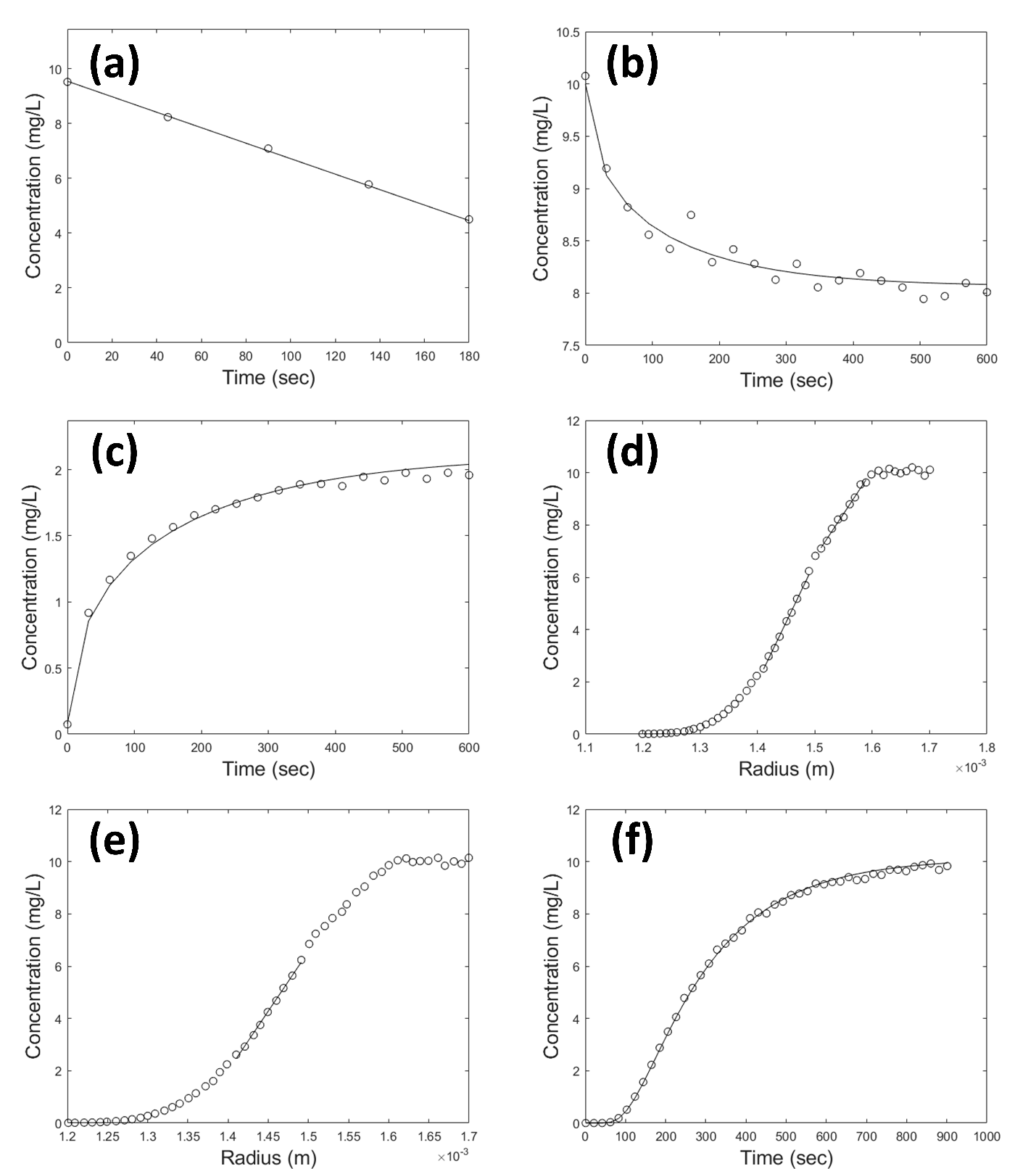 |
| --- |
| **Figure S1. Typical simulated datasets for the six methods included in the random error analysis. The circles represent the experimental data, while the line represent the model fit to the data. The simulated datasets are comparable to a dataset that an experimentalist might obtain with the given method. (a) Steady-state reaction, (b) Transient uptake of a non-reactive solute, (c) Transient release of a non-reactive solute, (d) Steady-state concentration profiles inside and outside a granule, (e) Steady-state reaction with concentration profile inside a granule, (f) Transient penetration of a solute to the centre of a granule.** |

- 1. **Calculation method for systematic error analysis**
     1. **Solute sorption**

In transient measurements, a solute can bind to the granule and introduce an error. We considered a solute that sorbs reversibly to the biofilm according to a linear isotherm:

| $S=k\cdot C,$ | (S9) |
| --- | --- |

where S is the adsorbed concentration (mass per non-water volume of the biofilm), k is the partition coefficient between the aqueous and biofilm phases, and C is the aqueous concentration. A mass balance over a granule section that considers both the dissolved and adsorbed component, in which only the dissolved solute diffuses, gives rise to the following equation (Stewart, 1996):

| $\left[ \epsilon_{W}+\left( 1-\epsilon_{W} \right)k \right]\frac{\partial C}{\partial t}=D_{Granule}\left( \frac{\partial^{2}C}{\partial r^{2}}+\frac{2}{r}\frac{\partial C}{\partial r} \right),$ | (S10) |
| --- | --- |

where ε_W_ is the volume fraction occupied by water. The mass balance in the case of no adsorption is as follows:

| $\epsilon_{W}\frac{\partial C}{\partial t}=D_{Granule}\left( \frac{\partial^{2}C}{\partial r^{2}}+\frac{2}{r}\frac{\partial C}{\partial r} \right),$ | (S11) |
| --- | --- |

If adsorption is not considered in the analysis of experimental data, the systematic error will thus be given by:

| $\frac{D_{Obs}}{D_{True}}=\frac{\epsilon_{W}}{\epsilon_{W}+\left( 1-\epsilon_{W} \right)k}$ | (S12) |
| --- | --- |

Table S2 lists the adsorption isotherms for different solutes and biofilms that were used in the calculation, based on experiments of Bassin, Pronk, Kraan, Kleerebezem, and Van Loosdrecht (2011), Kennedy, Lu, and Mohn (1992), Wang et al. (2019), and Beuling, van den Heuvel, and Ottengraf (2000). Adsorption data is generally reported per gVSS, thus a conversion was made where necessary, with a cell density of 350 gVSS/L (Stewart, 1998) and a water fraction of 0.8.

**Table S2. Linear adsorption isotherms for different solutes and biomass types in L/gVSS. The units follow from the linear relation between equilibrium concentration (mg/L) and the amount of solute adsorbed (mg/gVSS).**

| **Solute** | **Biomass type** | **Isotherm** | **Reference** |
| --- | --- | --- | --- |
|  |  | **L/gVSS** |  |
| Ammonium | Activated Sludge | 0.0063 | Bassin et al. (2011) |
| Ammonium | Activated Sludge | 0.0039 | Bassin et al. (2011) |
| Ammonium | Aerobic Granular Sludge | 0.0375 | Bassin et al. (2011) |
| Ammonium | Aerobic Granular Sludge | 0.0200 | Bassin et al. (2011) |
| Ammonium | Anammox Granular Sludge | 0.0046 | Bassin et al. (2011) |
|  |  |  |  |
| 2-chlorophenol | Anaerobic granular sludge | 0.0004 | Kennedy et al. (1992) |
| 3-chlorophenol | Anaerobic granular sludge | 0.0321 | Kennedy et al. (1992) |
| 4-chlorophenol | Anaerobic granular sludge | 0.0258 | Kennedy et al. (1992) |
| 2,3-dichlorophenol | Anaerobic granular sludge | 0.0229 | Kennedy et al. (1992) |
| 2,4-dichlorophenol | Anaerobic granular sludge | 0.0818 | Kennedy et al. (1992) |
| 2,5-dichlorophenol | Anaerobic granular sludge | 0.0345 | Kennedy et al. (1992) |
| 2,6-dichlorophenol | Anaerobic granular sludge | 0.0170 | Kennedy et al. (1992) |
| 3,4-dichlorophenol | Anaerobic granular sludge | 0.0429 | Kennedy et al. (1992) |
| 3,5-dichlorophenol | Anaerobic granular sludge | 0.0084 | Kennedy et al. (1992) |
|  |  |  |  |
| 2-nitrophenol | Anaerobic granular sludge | 0.0191 | Karim and Gupta (2002) |
| 4-nitrophenol | Anaerobic granular sludge | 0.0233 | Karim and Gupta (2002) |
| 2,4-nitrophenol | Anaerobic granular sludge | 0.0265 | Karim and Gupta (2002) |
|  |  |  |  |
| Dimethyl phthalate | River biofilm | 0.0060 | Wang et al. (2019) |
|  | River biofilm | 0.0058 | Wang et al. (2019) |
|  | River biofilm | 0.0072 | Wang et al. (2019) |
|  | River biofilm | 0.0035 | Wang et al. (2019) |
| Dibutyl phthalate | River biofilm | 0.0065 | Wang et al. (2019) |
|  | River biofilm | 0.0071 | Wang et al. (2019) |
|  | River biofilm | 0.0079 | Wang et al. (2019) |
|  | River biofilm | 0.0043 | Wang et al. (2019) |
| Di(2-ethylexyl)phtalate | River biofilm | 0.0083 | Wang et al. (2019) |
|  | River biofilm | 0.0204 | Wang et al. (2019) |
|  | River biofilm | 0.0200 | Wang et al. (2019) |
|  | River biofilm | 0.0089 | Wang et al. (2019) |
|  |  |  |  |
| Oxygen | Artificial biofilm | 0.0160 | Beuling et al. (2000) |

- - 1. **Deactivation procedures**

For most of the methods, deactivation of the biofilm is required if a reactive solute is used. To evaluate the impact that the deactivation procedure can have on the diffusion coefficient, we used the model of Westrin and Axelsson (1991). This model allows the calculation of the overall diffusion coefficient of a granule based on the granule structure (pore volume, EPS volume, cell volume) and diffusivity inside the microbial cells. The model is formulated as follows:

| $\frac{D_{eff}}{\mathfrak{D}_{aq}}=\left( \frac{D_{eff}}{D_{eo}} \right)\left( \frac{D_{eo}}{\mathfrak{D}_{aq}} \right),$ | (S13) |
| --- | --- |

where D_eff_ is the effective diffusive permeability of the biofilm, D_aq_ is the diffusion coefficient in water, and D_eo_ is the effective diffusive permeability of the extracellular matrix. The first term describes the effect of the bacterial cells and the second term describes the effect of the EPS. The first term is predicted with Maxwell’s equation for a suspension of permeable spheres (Chresand, Dale, Hanson, & Gillies, 1988):

| $\frac{D_{eff}}{D_{eo}}=\frac{2\frac{\mathfrak{D}_{aq}}{D_{C}}+\frac{\mathfrak{D}_{aq}}{D_{eo}}-2\epsilon_{C}\left( \frac{\mathfrak{D}_{aq}}{D_{C}}-\frac{\mathfrak{D}_{aq}}{D_{eo}} \right)}{2\frac{\mathfrak{D}_{aq}}{D_{C}}+\frac{\mathfrak{D}_{aq}}{D_{eo}}+\epsilon_{C}\left( \frac{\mathfrak{D}_{aq}}{D_{C}}-\frac{\mathfrak{D}_{aq}}{D_{eo}} \right)},$ | (S14) |
| --- | --- |

where D_c_ is the effective diffusive permeability in the bacterial cells, and ε_C_ is the volume fraction occupied by the cells. The second term of equation S13 is predicted based on the EPS and cell content of the biofilm (Westrin & Axelsson, 1991):

| $\frac{D_{eo}}{\mathfrak{D}_{aq}}=\frac{\left( 1-\frac{\epsilon_{P}}{1-\epsilon_{C}} \right)^{3}}{\left( 1+\frac{\epsilon_{P}}{1-\epsilon_{C}} \right)^{2}},$ | (S15) |
| --- | --- |

where ε_P_ is the volume fraction occupied by EPS. The degree to which the deactivation procedure breaks open the cell membranes and permeabilizes the cells was simulated by varying the effective permeability in the bacterial cells (D_c_). This permeability ranged from 0 (the cells are intact and a solute cannot diffuse through) to the permeability of the extracellular matrix (D_eo_). A typical granule composition was set with a porosity of 0.8, a cell volume fraction (ε_C_) of 0.17, and an EPS volume fraction (ε_P_) of 0.03.

1. **Results & Discussion**
   1. **Diffusion coefficient distribution from random error analysis**

| 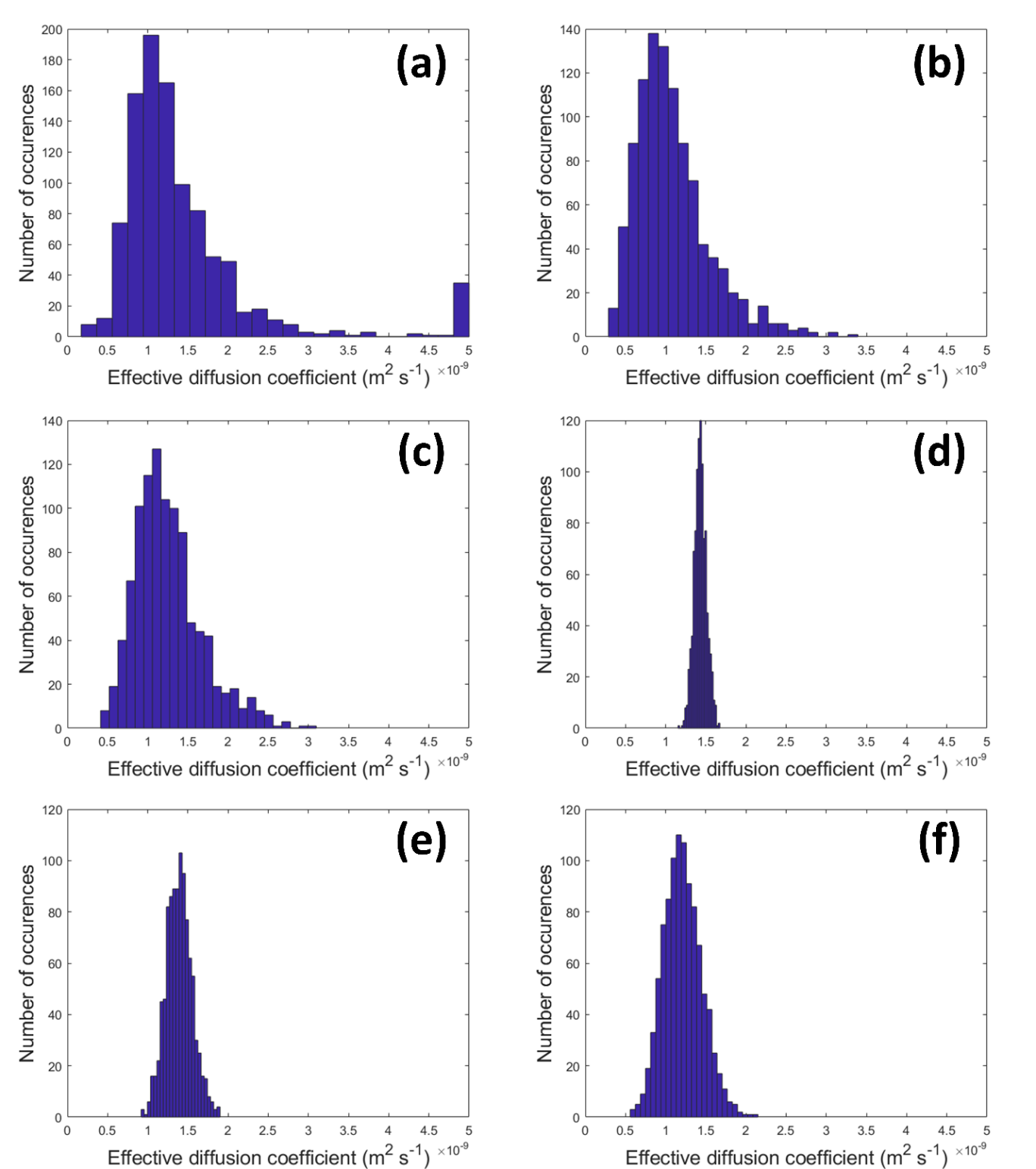 |
| --- |
| **Figure S2. Uncertainty analysis results: Diffusion coefficients obtained from Monte-Carlo simulations plotted as histograms. A total number of 1000 Monte-Carlo simulations were used. (a) Steady-state reaction, (b) Transient uptake of a non-reactive solute, (c) Transient release of a non-reactive solute, (d) Steady-state concentration profiles inside and outside a granule, (e) Steady-state reaction with concentration profile inside a granule, (f) Transient penetration of a solute to the centre of a granule.** |

- 1. **Standardized regression coefficients from random error analysis**

**Table S3. Squared standardized regression coefficients (β_i_^2^) of linear models of the diffusion coefficient. For a linear model, the sum of β_i_^2^ should be equal to 1. Typically the sum of β_i_^2^ is equal to or less then the R^2^. (a) Steady-state reaction, (b) Transient uptake of a non-reactive solute, (c) Transient release of a non-reactive solute, (d) Steady-state concentration profiles inside and outside a granule, (e) Steady-state reaction with concentration profile inside a granule, (f) Transient penetration of a solute to the centre of a granule.**

|  |  | **(a)** | **(b)** | **(c)** | **(d)** | **(e)** | **(f)** |
| --- | --- | --- | --- | --- | --- | --- | --- |
|  | **R^2^** | 0.60 | 0.92 | 0.95 | 0.81 | 0.98 | 1.00 |
| **1** | **V_g_** | 0.0411 | 0.3120 | 0.5663 | - | 0.1902 | - |
| **2** | **V** | 0.0008 | 0.0101 | 0.0202 | - | 0.0063 | - |
| **3** | **C_B_** | 0.0001 | 0.3324 | 0.0027 | - | 0.0001 | 0.0051 |
| **4** | **r_micro_** | - | - | - | 0.2254 | 0.0270 | - |
| **5** | **r_g_** | 0.0649 | 0.2300 | 0.3689 | - | 0.7358 | 1.0020 |
| **6** | **C_X_** | 0.4883 | - | - | - | - | - |
| **7** | **K** | 0.0134 | - | - | - | - | - |
| **8** | **q_max_** | 0.0120 | - | - | - | - | - |
| **9** | **C_micro_** | - | - | - | 0.5653 | 0.0107 | 0.0003 |
| **Sum of β_i_^2^** | | 0.62 | 0.88 | 0.96 | 0.79 | 0.97 | 1.01 |

- 1. **Systematic errors: sensitivity results**

| 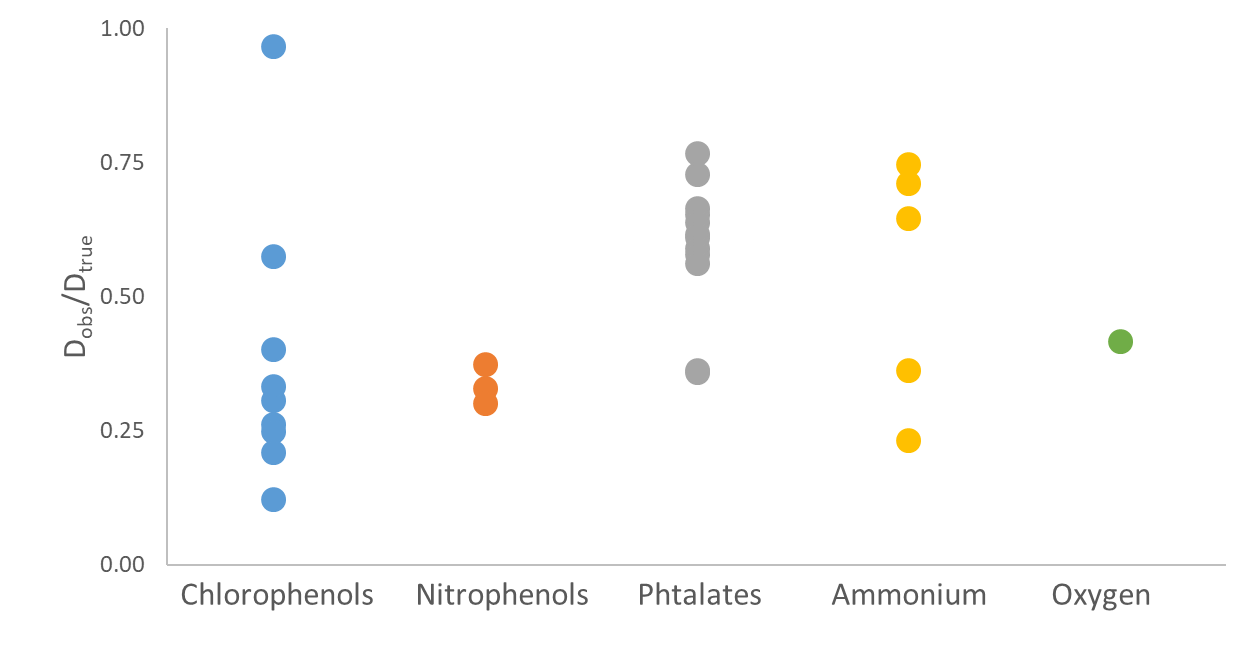 |
| --- |
| **Figure S3. Systematic error due to solute sorption for chlorophenols, nitrophenols, phthalates, ammonium, and oxygen in various types of biofilm. The systematic error was estimated based on reported linear isotherms or Freundlich isotherms that were approximated as linear isotherms.** |
|  |
| 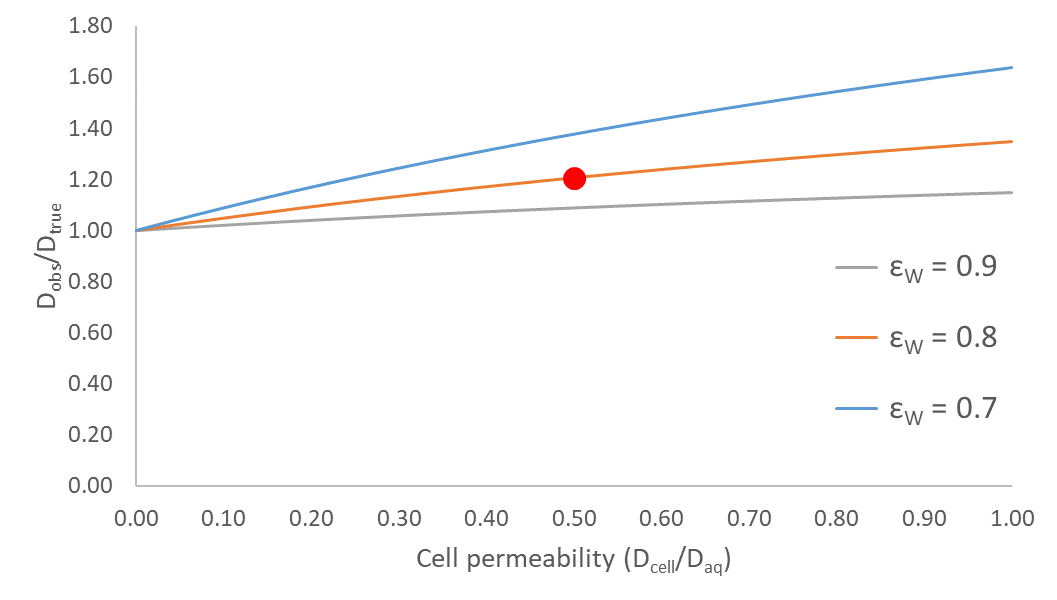 |
| **Figure S4. Systematic error as a result of cell permeabilization due to deactivation. The error is given for different granule water volume fractions (ε_W_). A cell permeability of 0 means that the cells are intact and completely impermeable to the solute, while a permeability of 1 means that the cells are completely permeable. As typical value, a cell permeability of 0.5 and a granule water volume fraction of 0.8 is used. The red dot denotes the typical value.** |
| 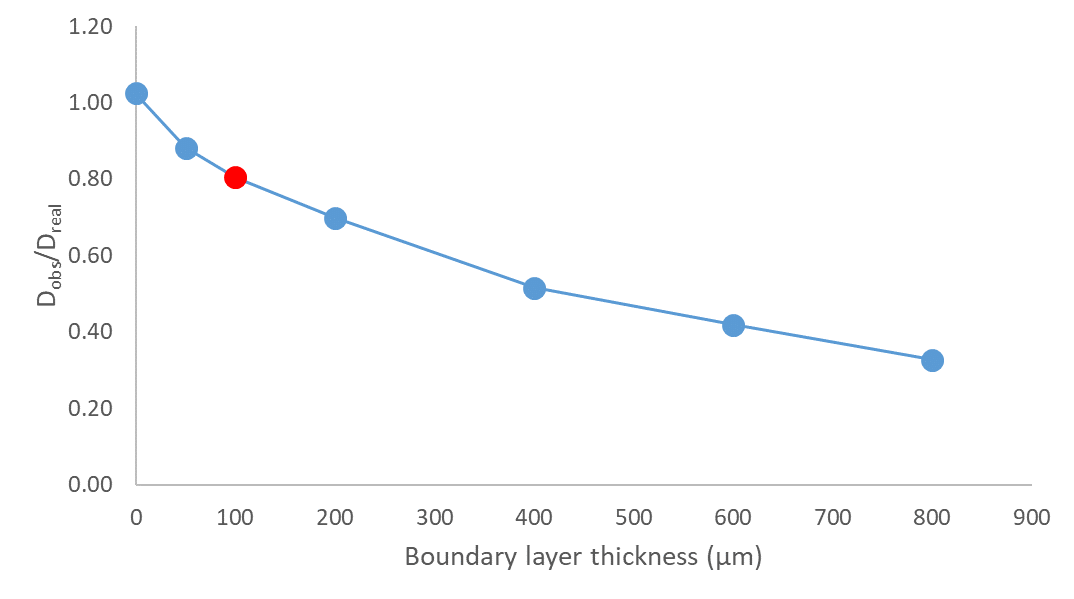 |
| **Figure S5. Systematic error due to the boundary layer, as function of the boundary layer thickness. Other granule parameters (as described in the main paper) are kept constant. The red dot denotes the typical value at a boundary layer thickness of 100 µm.** |
|  |
| 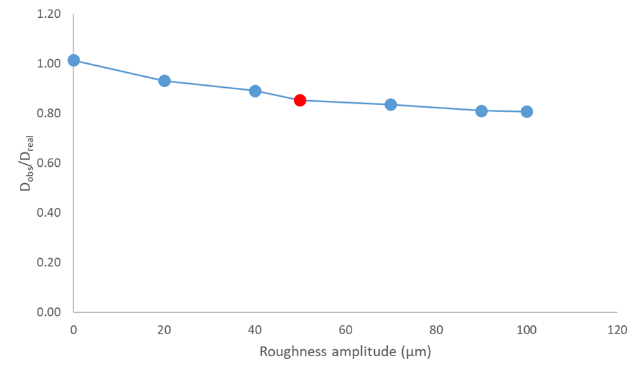 |
| **Figure S6. Systematic error from the surface roughness, as function of the roughness amplitude. The granule surface was described by a sine wave with 10 full sine waves over the entire granule radius. The roughness amplitude is the amplitude of the sine wave. An amplitude of 0 represents a smooth granule. The red dot denotes the typical value at a roughness amplitude of 50 µm.** |
| **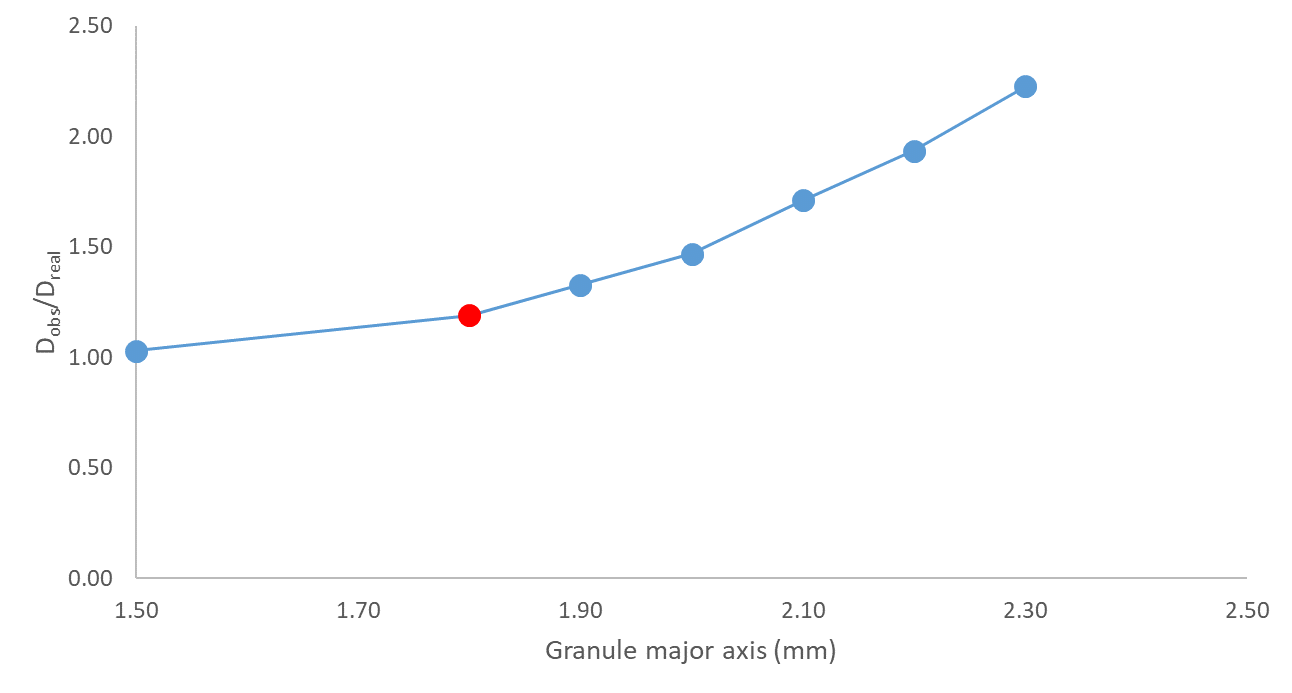** |
| **Figure S7. Systematic error as a result of granule sphericity, as function of major ellipse axis for an oblate spheroid. An increase in major axis length (compared to base case of 1.5mm) indicates a more ellipsoidal granule. The red dot denotes the typical value for a granule major axis of 1.80 mm, and a minor axis of 1.04 mm.** |
|  |
| **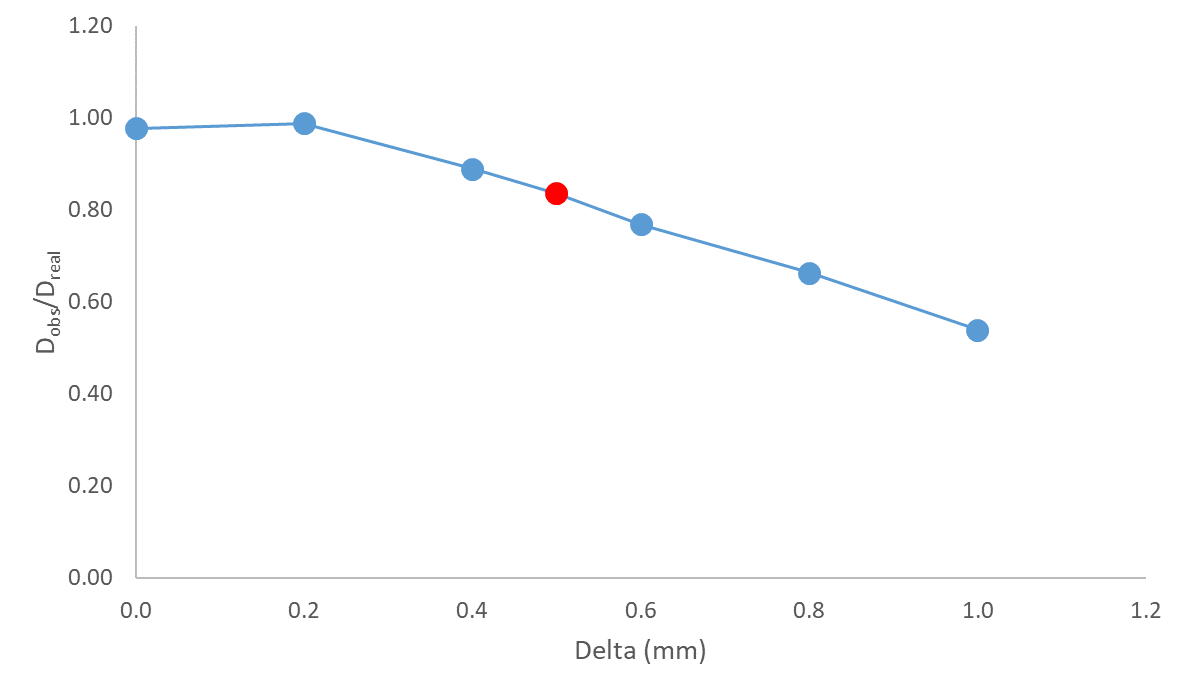** |
| **Figure S8. Systematic error through polydispersity, as function of polydispersity factor delta. This error was calculated with four granule sizes, two of which had the mean size (1.5 mm diameter), one was smaller by delta, and one was bigger by delta. The red dot denotes the typical value with resulting granule diameters of 1.0 mm, 1.5 mm, 1.5 mm, and 2.0 mm.** |

- 1. **Literature biofilm diffusion coefficients**

**Table S4. Diffusion coefficients in water and in a biofilm. Diffusion coefficient in water (D_aq_) are taken from (Stewart, 2003), with the exception of nitrate (Li & Gregory, 1974), nitrous oxide (Tamimi, Rinker, & Sandall, 1994), and phenol (Tang & Fan, 1987). The relative diffusion coefficient (f_D_) is the ratio of diffusion coefficient in a biofilm over diffusion coefficient in water, and is taken from Stewart (2003) as well. The biofilm diffusion coefficients are calculated from D_aq_ and f_D_. All values are for a temperature of 25 °C.**

| **Solute** | **D_aq_ (10^-9^ m^2^/s)** | **f_D_** | **D_bio_ (10^-9^ m^2^/s)** |
| --- | --- | --- | --- |
| Ammonium | 1.97 | 0.78 | 1.54 |
| Oxygen | 2.01 | 0.57 | 1.15 |
| Nitrous oxide | 1.88 | 0.61 | 1.15 |
| Nitrate | 1.90 | 0.66 | 1.25 |
| Acetate | 1.21 | 0.23 | 0.28 |
| Propionate | 1.06 | 0.31 | 0.33 |
| Butyrate | 0.87 | 0.20 | 0.17 |
| Phenol | 0.92 | 0.25 | 0.23 |
| Glucose | 0.67 | 0.30 | 0.20 |
| Sucrose | 0.52 | 0.19 | 0.10 |

- 1. **Diffusion sensitivity model**

The preliminary analysis of the sensitivity of the diffusion coefficient in biofilms models was based on a one dimensional diffusion-reaction equation in spherical coordinates:

| $D_{Granule}\left( \frac{\partial^{2}C}{\partial r^{2}}+\frac{2}{r}\frac{\partial C}{\partial r} \right)-q_{max}\frac{C}{K_{S}+C}C_{X}=0,$ | (S16) |
| --- | --- |

where *D_Granule_* is the diffusion coefficient (m^2^/s), *r* is the radial position in the granule (m), *q_max_* is the maximum uptake rate (1/s), *C* is the solute concentration within the granule (g/m^3^), *K_S_* the half-saturation coefficient (g/m^3^), and *C_X_* the biomass concentration (g/m^3^).

The equation applied to the granule as well as a mass transfer boundary layer (MTBL). The following boundary conditions were used: (1) zero flux at the granule core, (2) flux equality at the granule-MTBL interface, and (3) bulk liquid concentration at the MTBL-bulk liquid interface. The equation and boundary conditions were solved numerically in Matlab with lsqnonlin, a function for non-linear least squares fitting with constraints. A discretization scheme with central difference was applied, with a spatial stepsize of 10 µm. Input parameters were as follows: *r_G_* 1.5 mm, *D_Granule_* 1.2 · 10^-9^ m^2^/s, *D_aq_* 2.0 · 10^-9^ m^2^/s, *C_X_* 10,000 gCOD/m^3^, *q_max_* 3.54 gO_2_/gCOD/d, *K_S_* 0.2 g/m^3^, and d_MTBL_ 100 µm. The bulk liquid concentration was varied between 0.001 and 100 gO_2_/m^3^. This artificial range was chosen to simulate the transition from a barely penetrated granule (with concentrations below *K_S_*) to a fully penetrated one, while only changing a single parameter. A Monte Carlo approach was used with 1000 simulations for each bulk liquid concentration. For each Monte Carlo step, a diffusion coefficient was taken randomly from a normal distribution with D_Granule_ as its mean and a 0.12 · 10^-9^ m^2^/s standard deviation. The flux was calculated for each Monte Carlo step, which resulted in 1000 flux values per simulation. The sensitivity of the diffusion coefficient was evaluated by $\delta=\frac{\sigma_{Flux}}{\sigma_{DiffusionCoefficient}}$. A *δ* greater than 1 indicates a high sensitivity, while a *δ* smaller than 1 indicates a low sensitivity. The results can be seen in Figure S9.

| 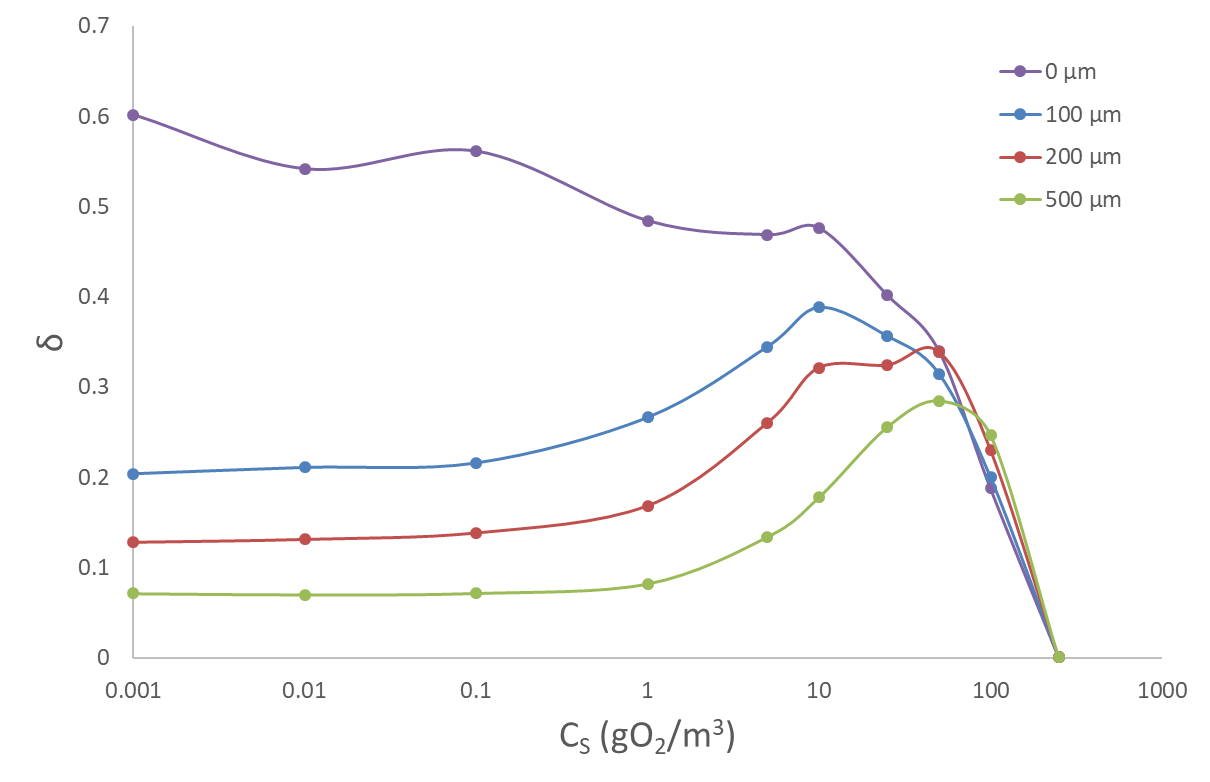 |
| --- |
| **Figure S9. Sensitivity of the diffusion coefficient (δ) as function of the bulk liquid substrate concentration for different mass transfer boundary layer thicknesses (0, 100, 200, and 500 µm thick). A lower δ indicates a lower sensitivity. For low bulk liquid concentrations (C_s_ < 1 g/m^3^) the sensitivity is reduced when a boundary is included. These low concentrations lead to surface reactions. For high bulk liquid concentrations (C_s_ > 100 g/m^3^) the sensitivity approach zero, as the granule becomes fully penetrated.** |

1. **References**

Bassin, J., Pronk, M., Kraan, R., Kleerebezem, R., & Van Loosdrecht, M. (2011). Ammonium adsorption in aerobic granular sludge, activated sludge and anammox granules. *Water Research, 45*(16), 5257-5265. doi:10.1016/j.watres.2011.07.034

Beuling, E. E., van den Heuvel, J. C., & Ottengraf, S. P. P. (2000). Diffusion coefficients of metabolites in active biofilms. *Biotechnology and Bioengineering, 67*, 53-60. doi:10.1002/(SICI)1097-0290(20000105)67:1<53::AID-BIT6>3.0.CO;2-H

Chresand, T. J., Dale, B. E., Hanson, S. L., & Gillies, R. J. (1988). A Stirred Bath Technique for Diffusivity Measurements in Cell Matrices. *Biotechnology and Bioengineering, 32*(8). doi:10.1002/bit.260320810

Crank, J. (1975). *The mathematics of diﬁusion* (2nd ed.).

Cronenberg, C. C. H., & Van Den Heuvel, J. C. (1991). Determination of glucose diffusion coefficients in biofilms with micro-electrodes. *Biosensors & Bioelectronics, 6*(3), 255-262. doi:10.1016/0956-5663(91)80011-L

Horn, H., & Morgenroth, E. (2006). Transport of oxygen, sodium chloride, and sodium nitrate in biofilms. *Chemical Engineering Science, 61*, 1347-1356. doi:10.1016/J.CES.2005.08.027

Karim, K., & Gupta, S. (2002). Biosorption of nitrophenols on anaerobic granular sludge. *Environmental Technology, 23*(12), 1379-1384. doi:10.1080/09593332508618443

Kennedy, K., Lu, J., & Mohn, W. W. (1992). Biosorption of chlorophenols to anaerobic granular sludge. *Water Research, 26*(8), 1085-1092. doi:10.1016/0043-1354(92)90144-S

Lewandowski, Z., Walser, G., & Characklis, W. G. (1991). Reaction kinetics in biofilms. *Biotechnology and Bioengineering, 38*(8), 877-882. doi:10.1002/bit.260380809

Li, Y.-H., & Gregory, S. (1974). Diffusion of ions in sea water and in deep-sea sediments. *Geochimica et Cosmochimica Acta, 38*(5), 703-714. doi:10.1016/0016-7037(74)90145-8

Stewart, P. S. (1996). Theoretical aspects of antibiotic diffusion into microbial biofilms. *J Antimicrobial agents chemotherapy, 40*(11), 2517-2522. doi:10.1128/Aac.40.11.2517

Stewart, P. S. (1998). A review of experimental measurements of effective diffusive permeabilities and effective diffusion coefficients in biofilms. *Biotechnology and Bioengineering, 59*, 261-272. doi:10.1002/(SICI)1097-0290(19980805)59:3<261::AID-BIT1>3.0.CO;2-9

Stewart, P. S. (2003). Diffusion in biofilms. *Journal of Bacteriology, 185*, 1485-1491. doi:10.1128/JB.185.5.1485-1491.2003

Tamimi, A., Rinker, E. B., & Sandall, O. C. (1994). Diffusion coefficients for hydrogen sulfide, carbon dioxide, and nitrous oxide in water over the temperature range 293-368 K. *Journal of Chemical and Engineering Data, 39*(2), 330-332.

Tang, W. T., & Fan, L. S. (1987). Steady state phenol degradation in a draft‐tube, gas‐liquid‐solid fluidized‐bed bioreactor. *AlChE Journal, 33*(2), 239-249. doi:10.1002/aic.690330210

Wang, L., Li, Y., Zhang, P., Zhang, S., Li, P., Wang, P., & Wang, C. (2019). Sorption removal of phthalate esters and bisphenols to biofilms from urban river: From macroscopic to microcosmic investigation. *Water Research, 150*, 261-270. doi:10.1016/j.watres.2018.11.053

Westrin, B. A., & Axelsson, A. (1991). Diffusion in gels containing immobilized cells: A critical review. *Biotechnology and Bioengineering, 38*(5), 439-446. doi:10.1002/bit.260380502
